# Supplementary material for: Adaptive federated filter for multi-sensor nonlinear system with cross-correlated noises
Source: PLoS One. 2021 Feb 19;16(2):e0246680. doi: 10.1371/journal.pone.0246680 (PMC7895398; doi:10.1371/journal.pone.0246680)
Supplement: Attachment — Submitted filename: Response to Reviewers.pdf [file pone.0246680.s002.pdf]

Dear Editors and Reviewers,

Thank you for your letter and for the reviewers' comments concerning our manuscript entitled "Adaptive Gaussian Federated Filter for Multi-sensor Nonlinear System with Same-paced Cross-correlated Noises" (ID: PONE-D-20-25635). Those comments are all valuable and very helpful for revising and improving our paper, as well as the important guiding significance to our researches. We have studied comments carefully and have made correction which we hope meet with approval. Revised portion are highlighted in yellow. The main corrections in the paper and the responses to the reviewer's comments are as flowing

Responds to the reviewer's comments:

Reviewer #1:

Comment 1: The aim of this paper is to design adaptive filtering methods for systems with cross-correlative noises, but some related works are missing, such as "A novel outlier-robust kalman filtering framework based on statistical similarity measure", "A novel robust Student's t-based Kalman filter", "A new adaptive extended Kalman filter for cooperative localization", "A novel robust Gaussian–Student's t mixture distribution based Kalman filter", "A novel Kullback–Leibler divergence minimization-based adaptive student's t-filter", "Variational adaptive Kalman filter with Gaussian-inverse-Wishart mixture distribution", "A slide window variational adaptive Kalman filter", "Design of Gaussian approximate filter and smoother for nonlinear systems with correlated noises at one epoch apart".

Response 1: The aforementioned works are closely related to this study and are very enlightening. Therefore, the above works have been systematically described in the review of nonlinear filtering algorithm for single sensor under the background of non-standard noise in the third paragraph of the revised manuscript.

The details are as follows： In a single sensor system, the problem of filtering under non-standard noise has always been the focus of researchers. An improved adaptive student's t-filter for the filtering of the linear system in the context of independent non-Gaussian heavy-tailed noise was proposed in [24-26]. A pseudo-measurement noise was constructed to form a pseudo-observation equation for the filtering of one-step related noise in nonlinear systems, and on this basis, new Gaussian approximation filters and smoothers are derived [27]. A novel adaptive Kalman filter based on Variational Bayesian method and Gauss-Inverse-Wishart mixture distribution was proposed for the linear system filtering problem with unknown system state and observed noise covariance matrix [28]. Reference [29] further improved the above filter based on the approximation of slide Window State Vectors

based on the work in reference [28]. Nonlinear filtering under cross-correlation noise has become an important branch of filtering under non-standard noise.

The relevant references are as follows：

24. Huang Y, Zhang Y, Chambers JA. A novel Kullback–Leibler divergence minimization-based adaptive student's t-filter. IEEE Transactions on signal Processing. 2019;67(20):5417-32.
25. Huang Y, Zhang Y, Zhao Y, Chambers JA. A Novel Robust Gaussian–Student's t Mixture Distribution Based Kalman Filter. IEEE Transactions on signal Processing. 2019;67(13):3606-20.
26. Yulong Huang YZZW, Ning Li，Jonathon Chambers. A novel robust Student's t based Kalman filter. IEEE Transactions on Aerospace and Electronic Systems. 2017;53(3):1545-54.
27. Huang Y, Zhang Y, Li N, Shi Z. Design of Gaussian approximate filter and smoother for nonlinear systems with correlated noises at one epoch apart. Circuits, Systems, and Signal Processing. 2016;35(11):3981-4008.
28. Huang Y, Zhang Y, Shi P, Chambers J. Variational Adaptive Kalman Filter with Gaussian-inverse-Wishart Mixture Distribution. IEEE Transactions on Automatic Control. 2020.
29. Huang Y, Zhu F, Jia G, Zhang Y. A Slide Window Variational Adaptive Kalman Filter. IEEE Transactions on Circuits and Systems II: Express Briefs. 2020.

Comment 2: Please explain that how to get the additional term in Eq (7)?

Response 2: The derivation of formula (7) in the original text is not clear enough, and the revised content is as follows

According to the observation equation in Equation (1) $\boldsymbol{z}_{m,k}=\mathbf{h}_{m,k}\left(\boldsymbol{x}_k\right)+\boldsymbol{v}_{m,k}$, one can get $\boldsymbol{z}_{m,k}-\mathbf{h}_{m,k}\left(\boldsymbol{x}_k\right)-\boldsymbol{v}_{m,k}=0$, and then $\boldsymbol{D}_{m,k}\boldsymbol{R}_{m,k}^{-1}\left[\boldsymbol{z}_{m,k}-\mathbf{h}_{m,k}\left(\boldsymbol{x}_k\right)-\boldsymbol{v}_{m,k}\right]=0$. Obviously, the substitution of this additional term into the process equation of Equation (1) will not change the original equation relationship in the process equation. By introducing an additional term $\boldsymbol{D}_{m,k}\boldsymbol{R}_{m,k}^{-1}\left[z_{m,k}-h_{m,k}\left(\boldsymbol{x}_k\right)-\boldsymbol{v}_{m,k}\right]$, the state equation in system (1) can be rewritten as follows.

$$\begin{aligned}\boldsymbol{x}_{k+1}&=f_k\left(\boldsymbol{x}_k\right)+\boldsymbol{w}_k+\boldsymbol{D}_{m,k}\boldsymbol{R}_{m,k}^{-1}\left[\boldsymbol{z}_{m,k}-h_{m,k}\left(\boldsymbol{x}_k\right)-\boldsymbol{v}_{m,k}\right]\\&=f_k\left(\boldsymbol{x}_k\right)+\boldsymbol{D}_{m,k}\boldsymbol{R}_{m,k}^{-1}\left[\boldsymbol{z}_{m,k}-h_{m,k}\left(\boldsymbol{x}_k\right)\right]+\boldsymbol{w}_k-\boldsymbol{D}_{m,k}\boldsymbol{R}_{m,k}^{-1}\boldsymbol{v}_{m,k}\end{aligned}\tag{7}$$

Comment 3: What are the technical challenges in devising these presented filters?

Response 3: Two major challenges are as follows

(1) How to replace the local filter in the federated filter with the nonlinear filter in the background of correlated noise and make it run normally;
(2) Proof of equivalence between AFF1-CN and AFF2-CN.

Comment 4: Please explain the differences between adaptive federated filter 1 for cross-correlated noises (AFF1-CN), adaptive federated filter 2 for cross-correlated noises(AFF2-CN) and high degree cubature federated filter for cross-correlated noises(HCFF-CN) essentially, and point out the advantages of AFF1-CN and AFF2-CN over HCFF-CN?)

Response 4:

1. There are two main differences between AFF1-CN, AFF2-CN and HCFF-CN for the design of nonlinear systems under the background of simultaneous cross-correlation noise, just as follows

(1) The local filters used by the three algorithms are different.

AFF1-CN uses a de-correlation framework as local filter, AFF2-CN is designed with CGAF[33] as subfilter, and HCFF-CN used GASF [30] as local filter. The mechanism of each filter to overcome the influence of cross-correlation noise on the filtering performance is different.

30. Wang X , Liang Y , Pan Q , et al. A Gaussian approximation recursive filter for nonlinear systems with correlated noises. Automatica, 2012, 48(9):2290-2297.
33. Huang, Y.L.; Zhang, Y.G.; Wu, Z.M.; Li, N.; Chambers, J.A. A novel adaptive Kalman fifilter with inaccurate process and measurement noise covariance matrices. IEEE Trans. Autom. Control 2018, 63, 594-601.

Firstly, the de-correlation framework constitute a new process function $F_{m,k}(\boldsymbol{x}_k)$ and process noise $\hat{\boldsymbol{w}}_{m,k}$ by adding additional term $\boldsymbol{D}_{m,k}\boldsymbol{R}_{m,k}^{-1}\left[\boldsymbol{z}_{m,k}-\mathbf{h}_{m,k}(\boldsymbol{x}_k)-\boldsymbol{v}_{m,k}\right]$ in the equation of state, and the new process noise $\hat{\boldsymbol{w}}_{m,k}$ is not related to the observed noise $\boldsymbol{v}_{m,k}$. Consequently, the filtering problem of cross-correlation noise is transformed into the filtering problem of independent white noise.

GASF approximates two-step state posterior predicted probability density function $p(\boldsymbol{x}_{k+1}|\boldsymbol{Z}_{m,k-1})$ and one-step observed posterior predicted probability density function $p(\boldsymbol{z}_{m,k}|\boldsymbol{Z}_{m,k-1})$ by Gauss, so as to substitute the number of correlation $\boldsymbol{D}_{m,k}$ representing noise cross-correlation into filtering calculation, so that filtering results can reflect the influence of cross-correlation noise.

In CGAF, since the posterior probability density function of process noise $\boldsymbol{w}_k$ obeisance to Gaussian distribution, the maximum posterior estimation method is used to estimate process noise $\boldsymbol{w}_k$, and then the estimated value is substituted into the filtering equation, so that the filtering result can reflect the influence of cross-correlation noise.

(2) The three filters have different preconditions.

To solve de-correlation filter and AFF2-CN, we need to assume that $p(\boldsymbol{x}_{m,k}|\boldsymbol{Z}_{m,k-1})$ and $p(\boldsymbol{z}_{m,k}|\boldsymbol{Z}_{m,k-1})$ obey Gaussian distribution, while HFF-CN needs to satisfy not only the above assumption, but also the assumption that $p(\boldsymbol{x}_{m,k+1}|\boldsymbol{Z}_{m,k-1})$ follows Gaussian distribution.

2. The advantages of AFF1-CN and AFF2-CN over HCFF-CN?

(1) AFF1-CN and AFF2-CN are general filters for solving nonlinear filtering problems in the background of synchronous cross-correlation noise. The numerical approximation rules are more flexible than that of HFF-CN. HCFF-CN directly

approximates the Gaussian integral by using the fifth-degree cubature rule, while AFF1-CN and AFF2-CN can not only approximate by using the fifth-degree cubature rule, but also choose any numerical point-based approximation rule according to needs, such as the third-degree cubature rule, UT transform rule, second-order Stirling interpolation method or Gauss-Hermitage rule, and so on.

(2) For HCFF, to overcome the effects of cross-correlation noise, the local filter's state prediction loop is independent of the updated state correction, so the estimate of the statistical characteristic of predictive PDF $p(\boldsymbol{x}_k \mid \boldsymbol{Z}_{m,k\text{-}1})$, not considering the observation information of recent $\boldsymbol{z}_{m,k}$ to estimate correction effect, and only consider $\boldsymbol{Z}_{m,k\text{-}1}$ correction action. This obviously affects the filtering accuracy to some extent. In the local filter of AFF1-CN and AFF2-CN, the state prediction loop and the state update cycle are coupled, considering the modification of the system state prediction by the latest observed values.

Comment 5: What is the meaning of P_{m} which lies in line 94 in this paper ? Please check and correct it.

Response 5: A specific explanation has been given in the article, just as follows

where k is the discrete sample time index; $m = 1, 2, \dots N$ is the sensor index; $\boldsymbol{x}_k \in \mathbf{R}^n$ and $\boldsymbol{z}_{m,k} \in \mathbf{R}^{p_m}$ are the system state and the mth measurement vector at k respectively, where the superscript n is the dimension of the state vector, p_m is the observation vector dimension of the m sensor;

Reviewer #2:

Comment 1: The research significance of this paper should be further strengthened by combining with the practical application of cross-correlations between process noise and local measurement noise.

Response 1: Related content has been added to the instruction section, just as follows

In practical application, the problem of noise cross-correlation always exists. For example, in a target tracking system, there would be some cross-correlation between the process and measurement noises if both of them are dependent on the system state [19]. Also, discretization on real continuous fusion systems may cause the cross-correlation to the process and measurement noises [20].

19. Feng, J., Wang, Z., Zeng, M. Distributed Weighted Robust Kalman Filter Fusion for Uncertain Systems with Auto-correlated and Cross-Correlated Noises, Information Fusion, 2013,14(1), 78-86.

20. Tian T, Sun S, Li N., Multi-sensor information fusion estimators for stochastic uncertain systems with correlated noises. Information Fusion, 2016, 27: 126-137.

Comment 2: Eq.(38) should be not the cross-correlated covariance.

Response 2:

Eq.(38) is the error covariance of the process noise, not the cross-correlated covariance. Improper expression leads to misunderstanding. The original statement is amended as follows

The definition of process noise $\boldsymbol{w}_k$ and observation noise $\boldsymbol{v}_{m,k}$ is the same as Eq.(1), where $\boldsymbol{Q}_k$ satisfies

Comment 3: A practical example should be provided to validate the proposed method in practical application and illustrate the research significance.

Response 3:

The work of this paper focuses on the establishment of the theoretical framework and equivalence proof of the proposed algorithm, and the effectiveness and rationality of the proposed algorithm are proved by the simulation experiment of maneuvering target tracking. Similar simulation experiments have been successfully applied in literature [23,35-37], with good application effect. Considering the valuable opinions of reviewers, the proposed algorithm is planned to be applied to a simulation example of integrated navigation system. It is planned to use The NaveGo toolbox of MATLAB based on real and simulation data of IMU and GPS, respectively, to apply the proposed algorithm to the integrated navigation system of vehicle and aircraft, and carry out the validation experiment of the effectiveness of the algorithm.

23. Ge, Q., Xu, D., and Wen, C., Cubature Information Filters with Correlated Noises and Their Applications in Decentralized Fusion, Signal Processing, 2014, 94, 434-444.
35. Wang, L. and Wang, S., High Degree Cubature Federated Filter for Multisensor Information Fusion with Correlated Noises, Mathematical Problems in Engineering, 2016.
36. Arasaratnam, I. and Haykin, S., Cubature Kalman Filters, Automatic Control, IEEE Transactions on, 2009, 54, (6), 1254-1269.
37. Jia, B., Xin, M., and Cheng, Y., High-Degree Cubature Kalman Filter, Automatica, 2013, 49, (2), 510-518.

Comment 4: The literature review on nonlinear filtering and data fusion should be improved by considering the listed references.

Response 4: The listed works are so instructive that they have been included in the first paragraph of the literature review.

To solve such problems, the information fusion algorithm must be based on the nonlinear fusion mechanism. For example, in order to solve the filtering precision decline and instability problem caused by various factors in the practical application of the integrated navigation system based on the nonlinear model, [13-16] proposed several solutions based on the unscented Kalman filter.

13. Gao B, Gao S, Hu G, Zhong Y, Gu C. Maximum likelihood principle and moving horizon estimation based adaptive unscented Kalman filter. Aerospace Science and Technology. 2018;73:184-96.
14. Gao B, Hu G, Gao S, Zhong Y, Gu C. Multi-sensor optimal data fusion for INS/GNSS/CNS integration based on unscented Kalman filter. International Journal of Control, Automation and Systems. 2018;16(1):129-40.
15. Hu G, Gao B, Zhong Y, Gu C. Unscented kalman filter with process noise covariance estimation for vehicular INS/GPS integration system. Information Fusion. 2020;64:194-204.
16. Hu G, Ni L, Gao B, Zhu X, Wang W, Zhong Y. Model predictive based unscented Kalman filter for hypersonic vehicle navigation with INS/GNSS integration. IEEE Access. 2019;8:4814-23.
